# Osteoid osteoma of the hip: imaging features

**DOI:** 10.1007/s00256-020-03515-8

**Published:** 2020-06-19

**Authors:** Jacques Malghem, Frederic Lecouvet, Thomas Kirchgesner, Souad Acid, Bruno Vande Berg

**Affiliations:** Department of Radiology, Cliniques Universitaires Saint-Luc, UCLouvain, Avenue Hippocrate, 10, B-1200 Brussels, Belgium

**Keywords:** Osteoid osteoma, Hip, Imaging, Atypical presentations, Differential diagnosis, Recurrence

## Abstract

Osteoid osteoma (OO), a small bone tumor relatively common in young subjects, frequently involves the hip. In addition to typical findings, we emphasize unsuspected clinical and imaging features including painless OO causing limping gait, non-visibility of totally mineralized nidus, absence of hyperostosis or adjacent edema, and recurrence at distance from the initial location. We also discuss the option of medical treatment for some cases of deep hip locations.

## Introduction

Osteoid osteoma (OO) accounts for 10 to 15% of all benign bone tumors. It mostly affects young subjects, mainly between 5 and 25 years with a male predominance [1–4]. Occurrence in older subjects is not unusual, with OO in 6 to 9% of subjects aged over 40 years in large series [1, 5].

An OO is a small, highly vascularized bone lesion that contains variable proportions of osteoid and woven bone surrounded by osteoblasts which form irregular trabeculae interspersed with osteoclasts and numerous dilated vessels [1]. The tumor itself, the nidus, does not invade adjacent bone, but it induces hyperostosis and bone marrow edema [1, 2]. The presence of nerve fibers can be demonstrated by special stains close to the blood vessels around the nidus and in some cases within the nidus [6, 7].

The majority of OOs arise in the cortex of long bones, where the lesion is usually diaphyseal or metadiaphyseal. Epiphyseal OOs are rare [2]. About 10% of OOs are intra-articular, of which nearly half occurs in the hip [8]. The most common location in the hip is the femoral neck. This area is intra-articular as it is surrounded by the synovial cavity and joint capsule [2]. This intra-articular location results in atypical clinical signs and unusual characteristics on imaging [8–11].

We summarize common clinical and imaging features observed in patients with OO and we focus on less common features observed especially in hip location.

## Clinical symptoms

Almost invariably, patients with OO have pain. Pain is initially mild and inconstant, and may become more severe and persistent. Typically, the pain is more intense at night [2, 3, 12].

Pain relief can be obtained with aspirin or non-steroidal anti-inflammatory drugs (NSAIDs) in nearly three-quarters of the cases [2, 12]. The pain is thought to be caused by increased pressure stimulating nerve fibers, linked to an abnormally high prostaglandin concentrations in the lesion (up to 30 times higher or more) [13]. This hypothesis explains the effectiveness of aspirin and NSAIDs, which both inhibit prostaglandin synthesis [14]. The duration of pain before diagnosis varies from weeks to years, with an average duration of 10 or 15.6 months [2, 5, 14, 15]. In intra-articular OO, the average delay between onset of symptoms and diagnosis is more than 2 years [8, 16].

**Fig. 1:**
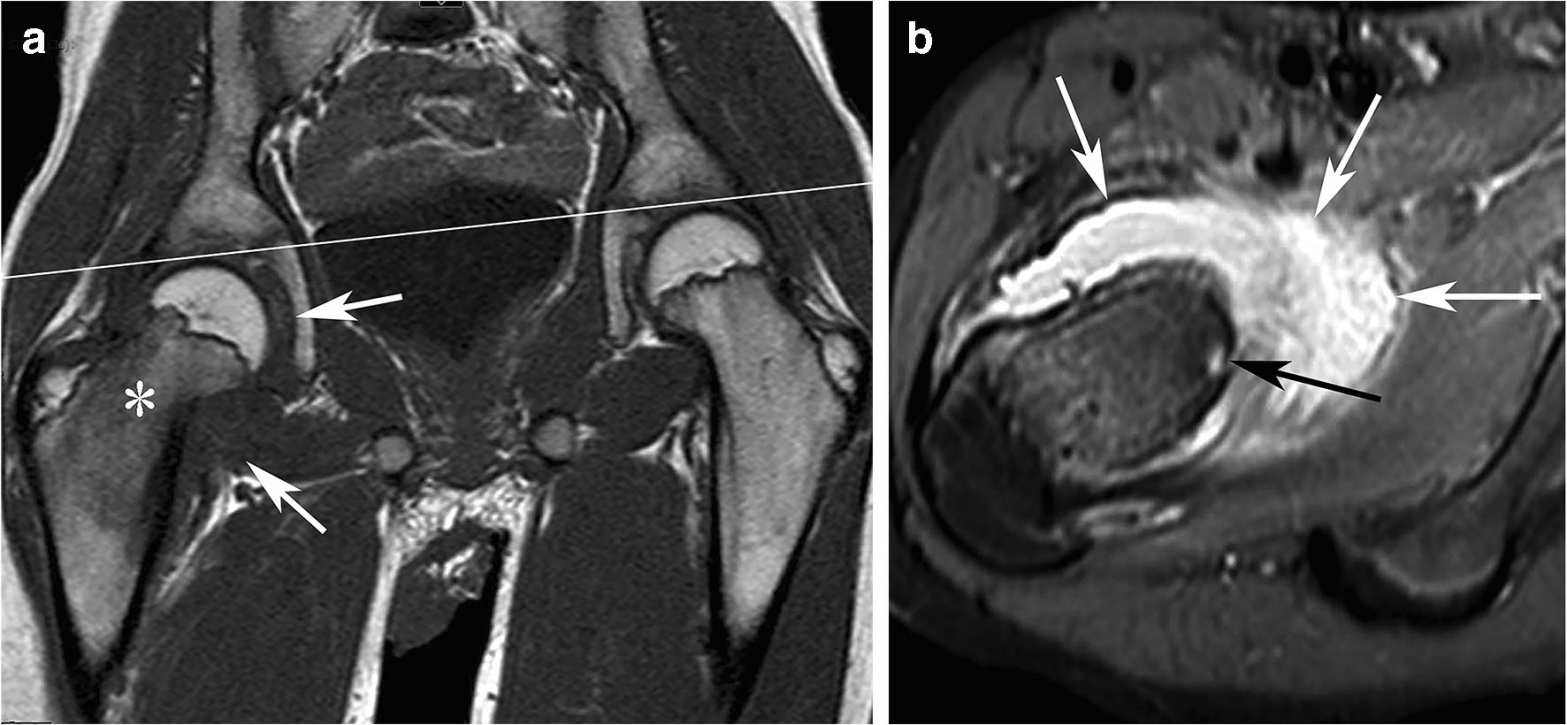
Painless OO in a 13-year-old boy with a painless limp and normal spinal MRI (not shown) requested for the limping gait. A coronal T1-weighted image of the pelvis (**a**) shows abduction of the right femur relative to the pelvic plane (line), low signal intensity in the bone marrow of the right femoral neck (asterisk), and synovial swelling with low signal intensity (arrows). An axial fat-saturated proton density-weighted MR image (**b**) shows both synovial swelling with high signal intensity (white arrows) and the OO nidus in the femoral neck (black arrow), which was confirmed by a CT scan (not shown). The OO was then successfully treated by radiofrequency ablation

However, painless OOs do exist with a few dozen of reported cases [17]. The absence of nerve fibers in the nidus has been suggested as a cause for painless OO [17]. The lesion may be detected by chance or due to thickening of bone or soft tissue when the lesion is close to the skin. As a matter of fact, half of painless OOs involves the phalanges [17]. Painless swelling may also precede the appearance of pain by several years in the case of OO located near the surface of the skin [18]. In asymptomatic patients with a deeply located OO, a functional symptom may be the presenting complaint. For hip OOs, a limping gait resulting from decreased range of motion due to joint effusion may be the presenting symptom (Fig. 1) [2, 19].

## Radiography and computed tomography

**Fig. 2:**
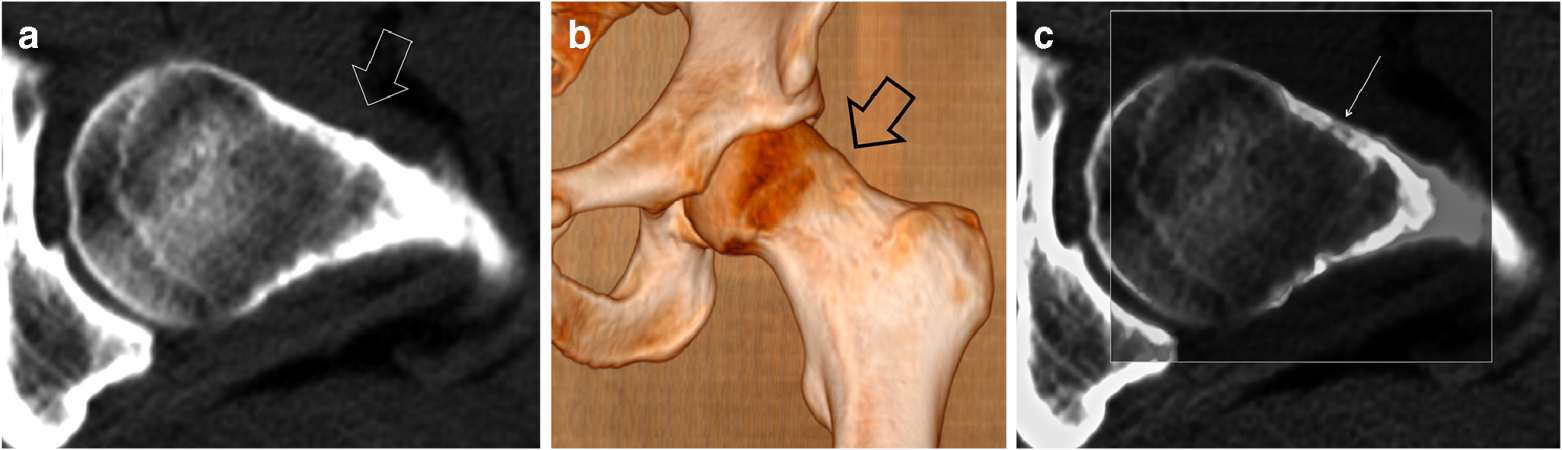
Almost normal CT appearance in a case of femoral neck OO in a 32-year-old man. Transverse CT image (**a**) shows minor cortical irregularities of the femoral neck (open arrow). 3D surface image (**b**) shows a bump in the head-neck junction (open arrow) suggestive of a femoroacetabular impingement syndrome. After surgical resection of that area, a typical nidus was found at microscopic examination within the resected fragment (not shown). A posteriori, a typical OO nidus can be seen on CT image after optimization of window width and level (arrow in **c**)

The nidus appears as a regular spherical or elliptical radiolucent area. Its diameter is generally less than 10 mm and very rarely more than 15–20 mm [2, 3, 20]. It is often poorly visible on radiographs, but it can be identified on computed tomography (CT) in almost all cases [21]. The nidus may present a calcified center (“bull’s-eye” appearance) [22, 23]. That is visible on CT in about 50% of cases [2, 21]. Their attenuation values are lower than those of cortical bone, i.e., 470 ± 222 Hounsfield units (HU) [24]. Rarely, the nidus is almost completely ossified and mimics a bone island [25] or normal cortical bone (Fig. 2).

**Fig. 3:**
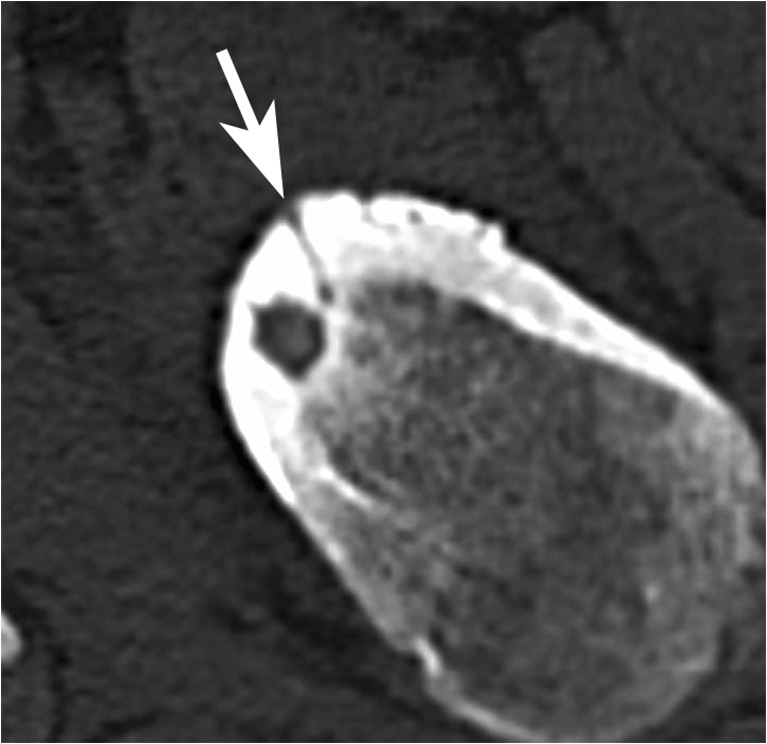
Axial CT image of a femoral neck OO demonstrates hypertrophic vascular channel (arrow) between the periosteal surface and the nidus area

CT images may depict thin linear or serpentine cortical radiolucencies connecting the nidus with the periosteal surface. These tunnels correspond to hypertrophic vascular channels (“CT vessel sign” or “vascular groove”) [26, 27] (Fig. 3). Their maximum diameter is about 1 mm, and these vascular grooves can be detected in about 80% of cases on high-resolution CT images [28].

Generally, cortical bone adjacent to the nidus is thickened. When present, periosteal reaction is generally solid, rarely exhibiting a multilayered appearance [2]. In the case of femoral neck OO, periosteal reaction may be absent. The femoral neck periosteum differs from that of the shaft and is unable to produce prominent cortical thickening (analogous to the lack of callus formation after intra-articular fractures of femoral neck) [2, 9, 15]. Reactive intra-articular cortical thickening absent or minimal is believed to due to a lack of cambium, the inner cellular layer of the periosteum [20]. The cellular periosteum of femoral neck surface is less than twofold compared to the femoral diaphysis [29]. However, an intra-articular OO can induce bone sclerosis at distance from its location, i.e., in the upper part of the underlying femoral shaft [2, 9, 23, 30].

When the OO is located in the lower section of the femoral neck, periosteal thickening can be more pronounced in the diaphysis than in the femoral neck. Therefore, the nidus should also be sought looked for in the proximal portion of the hyperostosis as it is not always located centrally with respect to the cortical thickening.

Often, OOs involving the cancellous bone do not produce reactive trabecular bone sclerosis [15, 21]. Therefore, hip OO involving the trabecular bone of the proximal femur or of the acetabulum can be difficult to diagnose due to limited bone sclerosis [2, 10, 21, 23, 31–33].

**Fig. 4:**
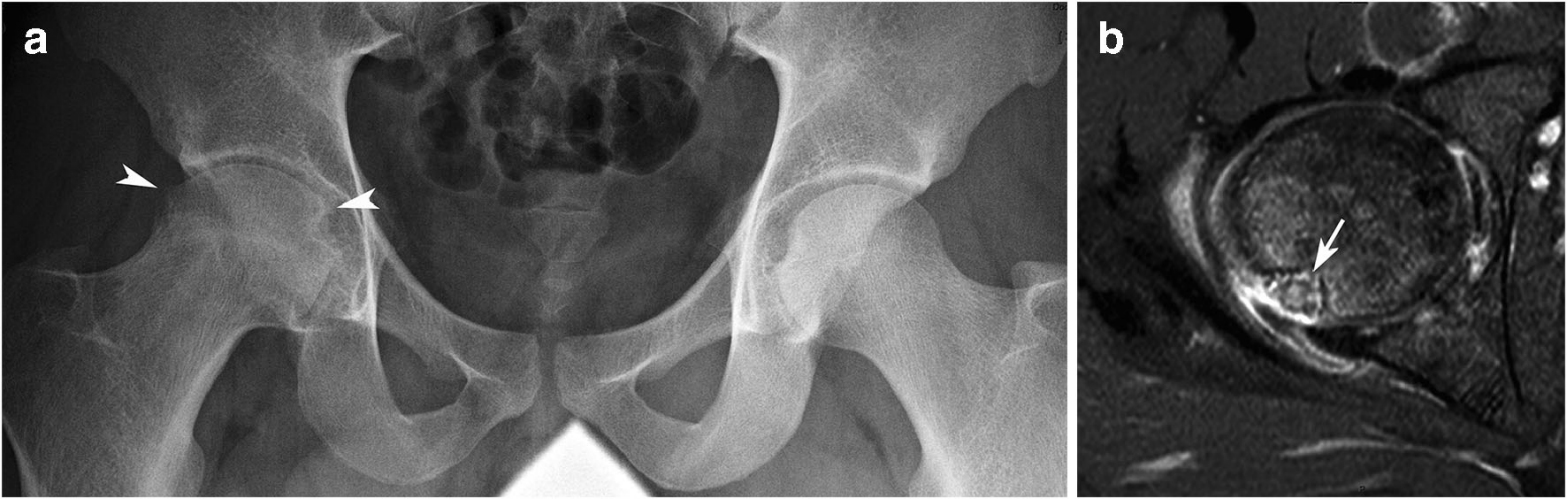
Femoral head OO mimicker of hip arthritis in a 19-year-old man with continuous right hip pain for 2 years despite drilling of the femoral head and an open synovial biopsy. **a** Pelvic radiograph demonstrates right hip osteoporosis with joint space narrowing and osteophytes (arrowheads) suggestive of chronic arthritis. Axial fat-saturated post-contrast T1-weighted MR image (**b**) demonstrated an OO in the posterior aspect of the femoral head (arrow)

Other regional bone changes may also be associated with hip OOs including regional osteoporosis, widening of the neck, coxa magna, widening or narrowing of the joint space, and osteophytes mimicking early osteoarthritis (Fig. 4) [2, 11, 15, 23, 33–35]. Hypertrophy of the head-neck junction due to stimulation of the physeal activity during growth can be observed in association with hip OO [11, 36, 37]. This hypertrophy of the head-neck junction may indirectly result in a diagnosis delay of the OO because the symptoms are wrongly attributed to a femoroacetabular impingement rather than to the OO (Fig. 2). Femoroacetabular impingement syndrome is currently the most common misdiagnosis in children and adolescents with hip OO [38].

## Magnetic resonance imaging

The diagnosis value of magnetic resonance imaging (MRI) for the OO is controversial because the nidus cannot be clearly detected in up to 35% of cases [10, 21]. However, some authors consider that this high frequency of MRI-occult nidus can be attributed to obtention of low special resolution images [16].

The nidus has low to intermediate signal intensity on T1-weighted images and variable signal intensity on T2-weighted images, depending of the amount of mineralization present in the center of the nidus [20]. After injection of a gadolinium chelate, the signal is usually moderately enhanced on T1-weighted MR images. The nidus is more conspicuous on fat-suppressed T2-weighted images and fat-suppressed T1-weighted gadolinium-enhanced images [16, 23].

**Fig. 5:**
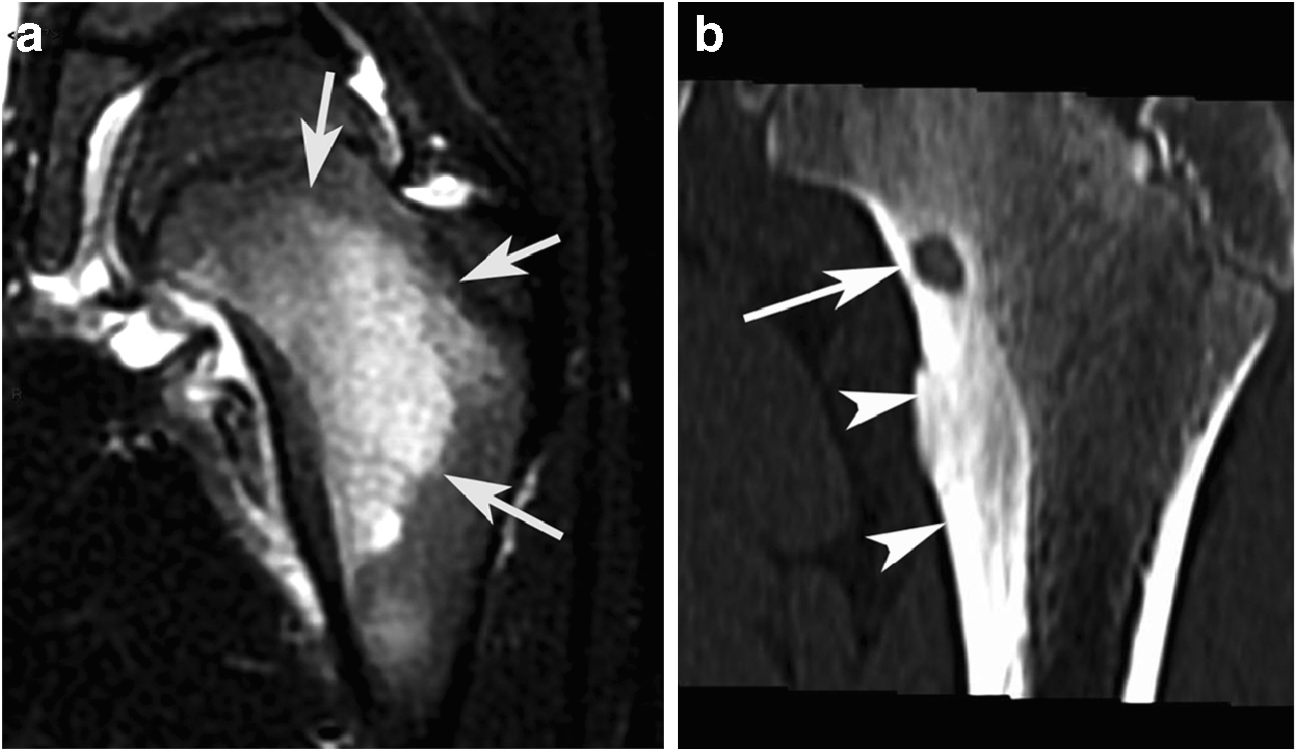
OO mimicker of stress fracture on MRI. Coronal fat-saturated T2-weighted image (**a**) shows a semicircular medullary edema-like pattern (arrows) adjacent to the medial cortex of the femoral neck, a frequent location for stress injuries. But a coronal CT image (**b**) reveals a typical OO nidus (arrow). Note important hyperostosis (arrowheads) below the OO but not on the femoral neck

Marrow and peri-osseous changes adjacent to the OO with high signal on fat-suppressed T2-weighted and gadolinium-enhanced T1-weighted images are considered to be invariably present [23]. Pathologic findings in the abnormal paraosseous soft tissues correspond to myxomatous changes associated with mild to moderate inflammatory cell infiltration. The medullary changes correspond to depleted cellular elements replaced by proteinaceous material [39]. This MRI findings may be referred to as “edema-like signal intensity” and “bone marrow edema-like signal intensity” [40]. In femoral neck OOs, this edema-like pattern may present a half-moon appearance with its base lying on the cortex (“half-moon sign”) (Fig. 5). Although some authors consider this sign to be very accurate [41], it may be observed in other conditions, stress fractures in particular, that tend to occur in the same location [42, 43]. Bone marrow and soft tissue changes can interfere with the diagnosis performance of MRI for OOs. When these changes are extensive, the nidus may be swamped in adjacent bone and soft tissues changes and cannot be recognized.

An absence of changes in soft tissue and bone marrow around an OO is noted by some authors (up to more than a third of cases of MRI without fat suppression), particularly in patients treated with salicylates or NSAIDs [21]. This possible effect is debated [16].

**Fig. 6:**
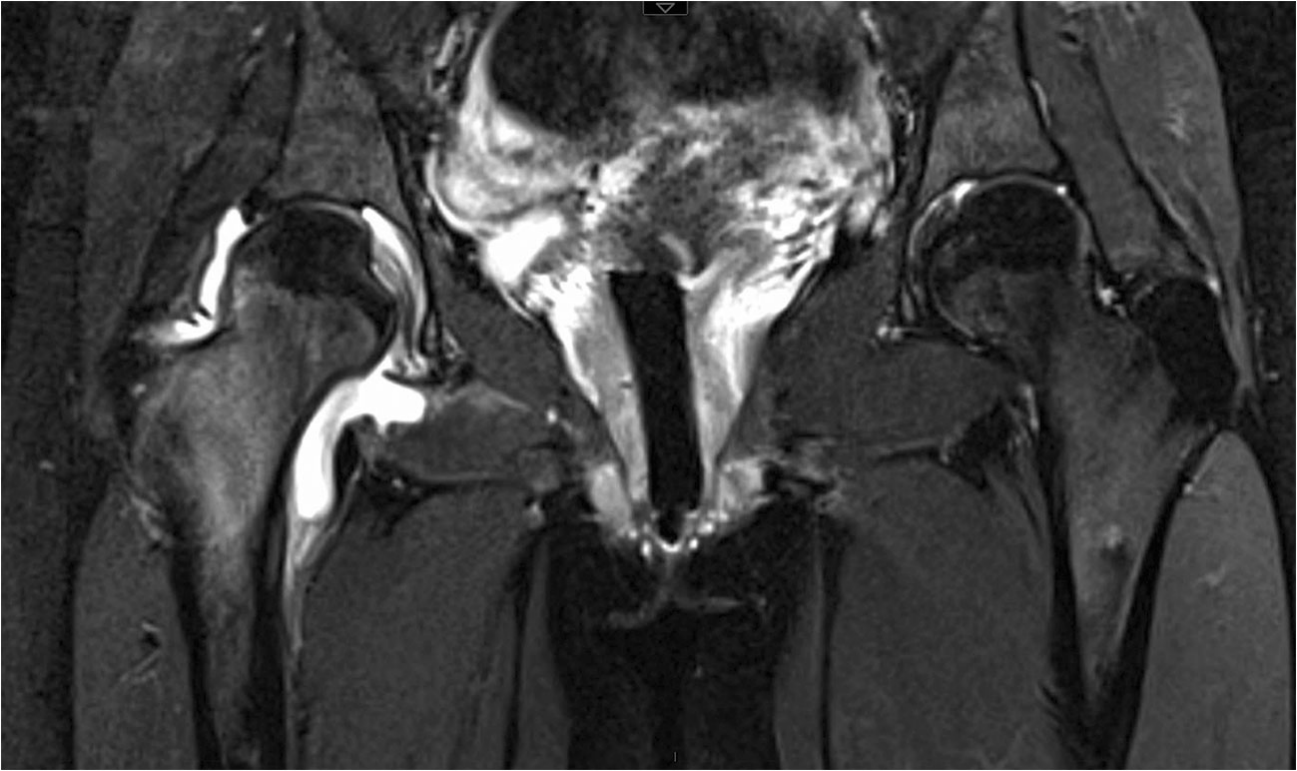
OO mimicker of arthritis. Coronal fat-saturated T2-weighted MR images of the pelvis shows synovial thickening and edema-like bone marrow in the right femoral neck. Hip arthritis had been diagnosed and treated with local steroid injections. A bone scintigraphy suggested a diagnosis of OO at the anterior aspect of the femoral neck, confirmed by a CT scan (not shown)

Intra-articular OO is associated with joint effusion, resulting from a non-specific proliferative synovitis usually lymphofollicular in nature [39]. This synovial reaction is a major diagnostic trap, because it mimics an inflammatory disease (Fig. 6).

**Fig. 7:**
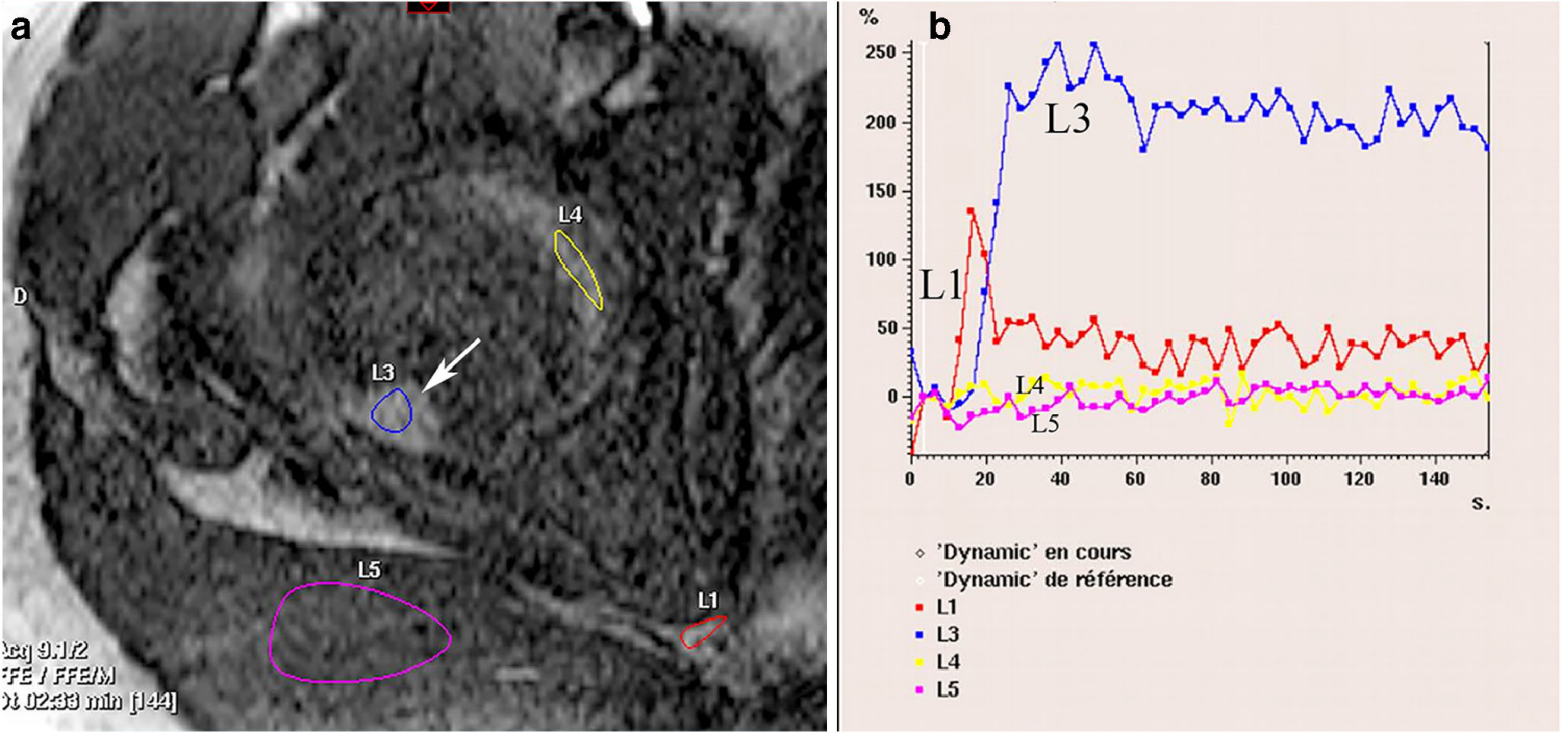
DCE-MRI of an OO. Axial post-contrast gradient-echo T1-weighted MR image of a femoral head OO (arrow in **a**) (same case as Fig. 4). Signal enhancement curves (**b**) show that the enhancement of the OO (L3) starts 3 s after that of the artery (L1) with a similar slope. Limited enhancement is measured in the joint cavity (L4) and in a muscle (L5)

Dynamic contrast-enhanced MR imaging (DCE-MRI) can contribute to increase the degree of diagnostic confidence of an OO [28]. Using sequences repeated every 30 s after the administration of gadolinium, Liu et al. showed that OOs exhibit a peak in signal enhancement during the arterial phase in 82% of the cases [44]. Several subsequent studies confirmed that this enhancement pattern was present in 82 to 100% of OOs using temporal resolutions that varied between 12 and 30 s [28, 45–48]. Some authors used a higher temporal resolution (sequences repeated every 3 s) and demonstrated that an enhancement delay of less than 6 s between the lesion and an adjacent artery was also a typical characteristic of OO (Fig. 7) [49]. Pottecher et al. used a variable time resolution and suggested that to eliminate the Nyquist limit, a temporal resolution of 3 s would be required [28]. These observations also support the use of angiographic imaging on 4D MRI [50].

**Fig.8:**
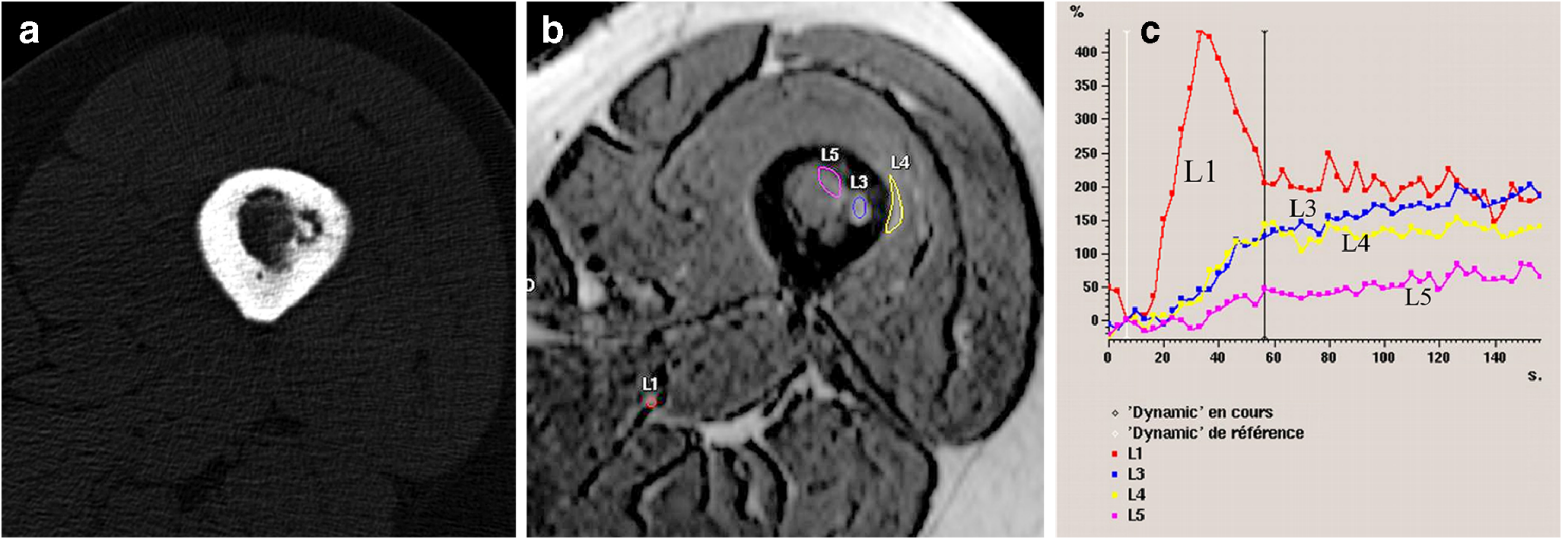
A femoral Brodie abscess on axial CT image (**a**) mimics an OO. However, DCE-MRI image and enhancement curves (**b** and **c**) show slow and limited enhancement in the abscess (L3) and adjacent soft tissue (L4) in comparison with a reference artery (L1). Signal enhancement in the abscess begins later than that in the artery in comparison with that expected in an OO

DCE-MRI is a very sensitive modality for the diagnosis of OOs. Its specificity varies on the lesions in the control group, since a similar peak in signal enhancement can be seen in other tumors. Its value in differentiating between OO and a Brodie abscess must be emphasized. Brodie abscess may mimic OO as the two lesions consist on small bone lesion with a calcified center and adjacent bone marrow and soft tissue changes. In a Brodie abscess, a post-contrast rim enhancement with a central non-enhanced area due to bone necrosis and pus may be present, while in OO enhancement is more diffuse [23]. On DCE-MRI, osteomyelitis enhances gradually without an early arterial peak unlike OO (Fig. 8) [28, 44, 51].

Similar results can be obtained by CT perfusion [52]. However, if possible, MRI should be favored over CT for radiation protection issues, especially for the pelvic region.

## Other imaging modalities

In clinical practice, CT is the best imaging modality to detect the OO nidus, and MRI is the best modality to recognize associated soft tissue and marrow changes [20]. However, other imaging methods may have been obtained in the workup of patients with hip OO and are worth mentioning.

Bone scintigraphy using technetium 99 m methylene diphosphonate (^99m^Tc-labeled bisphosphonates) can detect the presence of an OO of the hip, even in occult cases at radiography and MRI [10]. Tracer uptake is almost always increased, but the absorption can be hidden by the intense activity of a growing physeal plate when the OO is located immediately near this area [53]. Although uptake is not specific, the presence of a double density pattern with a focal increased uptake surrounded by a less dense uptake area may suggest diagnosis of OO [22, 23]. Tracer detection can be improved using a cross-sectional approach in single photon emission computed tomography (SPECT), enabling better 3D localization. The combination of a SPECT and a CT scan (SPECT-CT) can represent a “one-stop” imaging modality for OO since it combines the very high sensitivity of scintigraphy with the very high diagnostic specificity of CT [54]. Positron emission tomography (PET) using the ^18F^-FDG tracer may produce false-negative results in the detection of OO [55]. However, ^18^F-labeled sodium fluoride that is a bone-seeking radiotracer with uptake characteristics comparable to those of ^99m^Tc-labeled bisphosphonates can also provide an accurate diagnosis of OO [55].

**Fig. 9:**
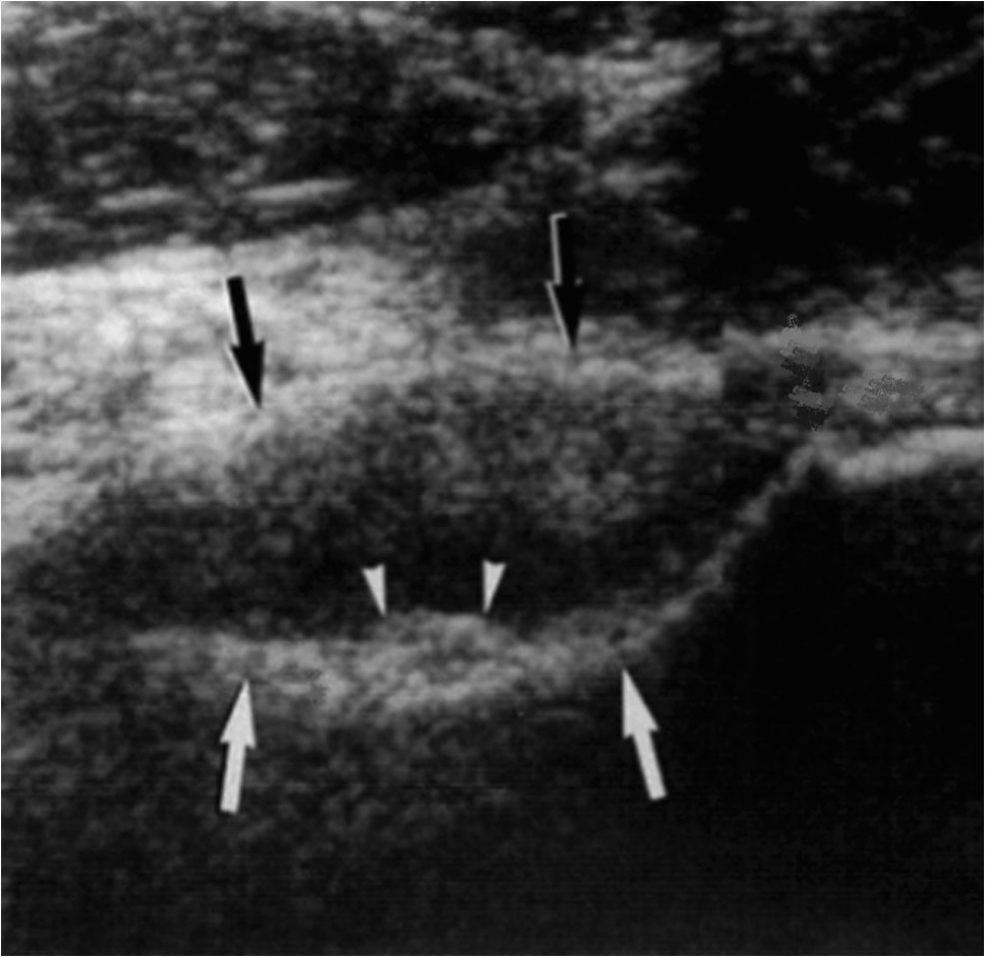
An ultrasound image of the hip shows synovial/capsular swelling (black arrows) as well as a small nodule with an echogenic surface (arrowheads) on the anterior aspect of the femoral neck (white arrows). This small nodule corresponded to a very superficial OO identified on CT (not shown) (from Malghem J et al. [57])

Ultrasound has very limited diagnostic value for OO. In the case of intra-articular OO, ultrasound can show synovitis leading to a false diagnosis of inflammatory disease [56]. If the OO is accessible, ultrasound can also show a cortical irregularity (Fig. 9) [56, 57].

## Recurrence after treatment

It is beyond the scope of this article to review the numerous therapeutic options. However, patients with treated OO may experience recurring symptoms and the question of OO residue or recurrence may arise.

Surgical excision has long been the gold standard in the treatment of OO. When OO is easily accessible and in case of diagnostic uncertainty requiring histological analysis, curettage remains a treatment option [58]. Recurrences of OOs after resection occur in 4.5% and in 12% after curettage [58]. Arthroscopic management of intra-articular OO may have a success rate exceeding 90% [59].

Currently, radiofrequency and laser therapy ablation are the most widely and validated used methods for treatment of OO [47]. Recently, new ablation technologies have been used to treat OOs, namely cryoablation [60]. The advantages over surgery include a lower invasiveness and a lower cost. Recurrence rates following percutaneous ablation vary between 2 and 27% [47], but they seem to decrease in some recent series [48, 61–63]. In a recent review by Lindquester et al., recurrent rate was 5.6% without significant difference when comparing radiofrequency ablation and cryoablation, and with a similar success rate for intra-articular lesions [60]. Recurrence is generally considered to result from incomplete excision, ablation, or destruction.

Response evaluation to percutaneous ablation is not straightforward. On CT, a persistent nidus on CT does not necessarily indicate treatment failure since successfully treated OOs remain unchanged or variably ossified [48, 64]. On conventional MRI, bone marrow edema and signal enhancement after injection of gadolinium may persist after successful thermal ablation [64]. On DCE-MRI, however, the persistence of an early, intense enhancement peak indicates treatment failure. Successfully treated OOs show slow or no enhancement [46, 47].

**Fig. 10:**
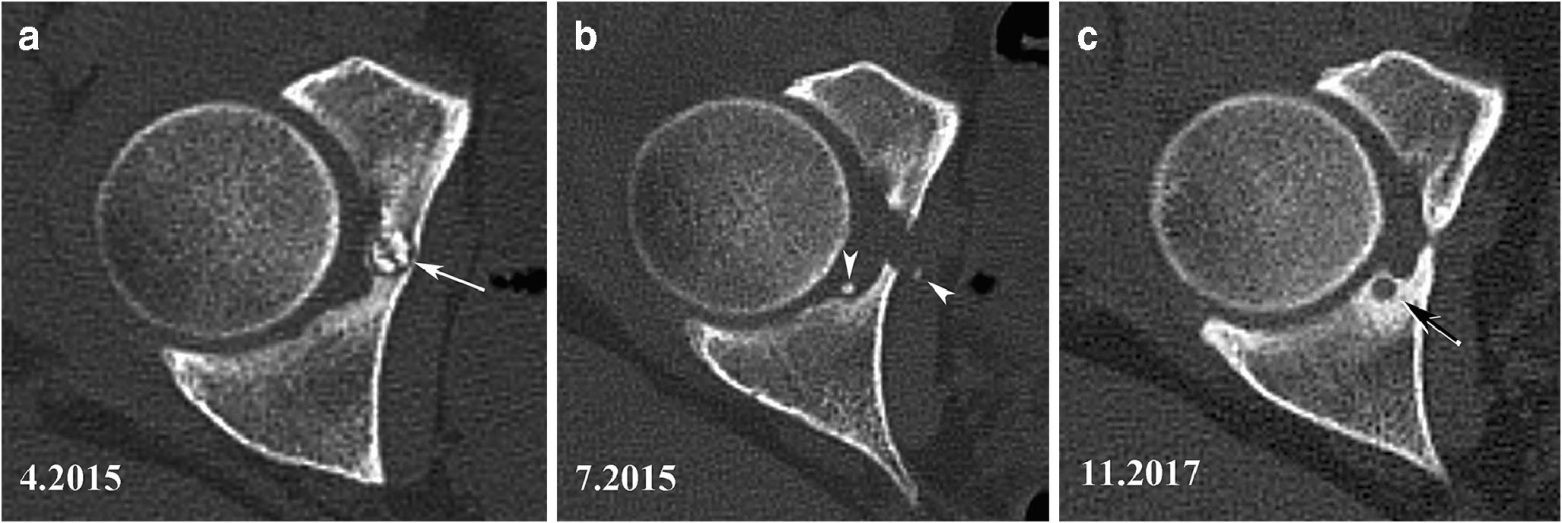
Recurrence near the original site of a resected OO. An OO involving the deeper aspect of the acetabulum (arrow in CT image in **a**) was arthroscopically resected. A CT examination performed after surgery (**b**) showed complete lesion resection and the presence of small bone fragments (arrowheads) near the resected site. The pain recurred after several months and repeated CT examination performed 30 months after surgery demonstrated appearance of a small lucent area surrounded by hyperostosis near the resection site (arrow in **c**). Recurring OO nidus was confirmed on DCE-MRI (not shown) and successfully electrocoagulated

**Fig. 11:**
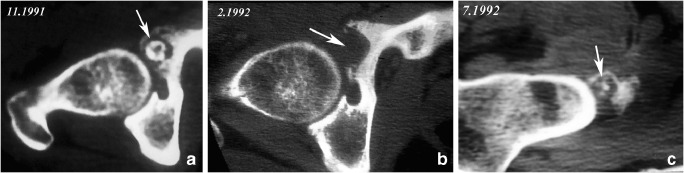
Soft tissue recurrence of an OO. Initial axial CT image shows an acetabular OO (arrow in **a**). Postoperative CT image (**b**) shows the large resection area of the lesion (arrow). Pain recurred at follow-up and a CT examination obtained several months after surgery demonstrated heterotopic ossification with a central nidus-like pattern (arrow in **c**) adjacent to the medial aspect of the femoral neck. Recurrent OO in soft tissue was demonstrated at microscopic analysis of the resected lesion

Due to the limited number of published cases of OO recurred after ablation, we ignore whether hip location is a risk factor or not. We observed two cases of hip OO recurrence at distance from their initial site. In one case, it was a completely resected acetabular OO by arthroscopic surgery which had recurred in bone at 1 cm of the initial site (Fig. 10). Implantation of tumor cells during surgical procedure is a plausible explanation. An OO recurrence in bone deeper than the initial site has also been reported after arthroscopic excision in another joint [65]. The other case was a completely surgically resected acetabular OO recurred in soft tissue 3 cm from the initial site. The new OO developed within postoperative periarticular heterotopic ossifications (Fig. 11). It may be hypothesized that a fragment of the initial lesion fell into the operative field and may have developed there inside the focus of heterotopic ossification. Although these two observations may seem anecdotal, they remind us the difficulty to treat some OOs in difficult-to-reach areas of the hip.

## Spontaneous resolution and medical treatment

**Fig. 12:**
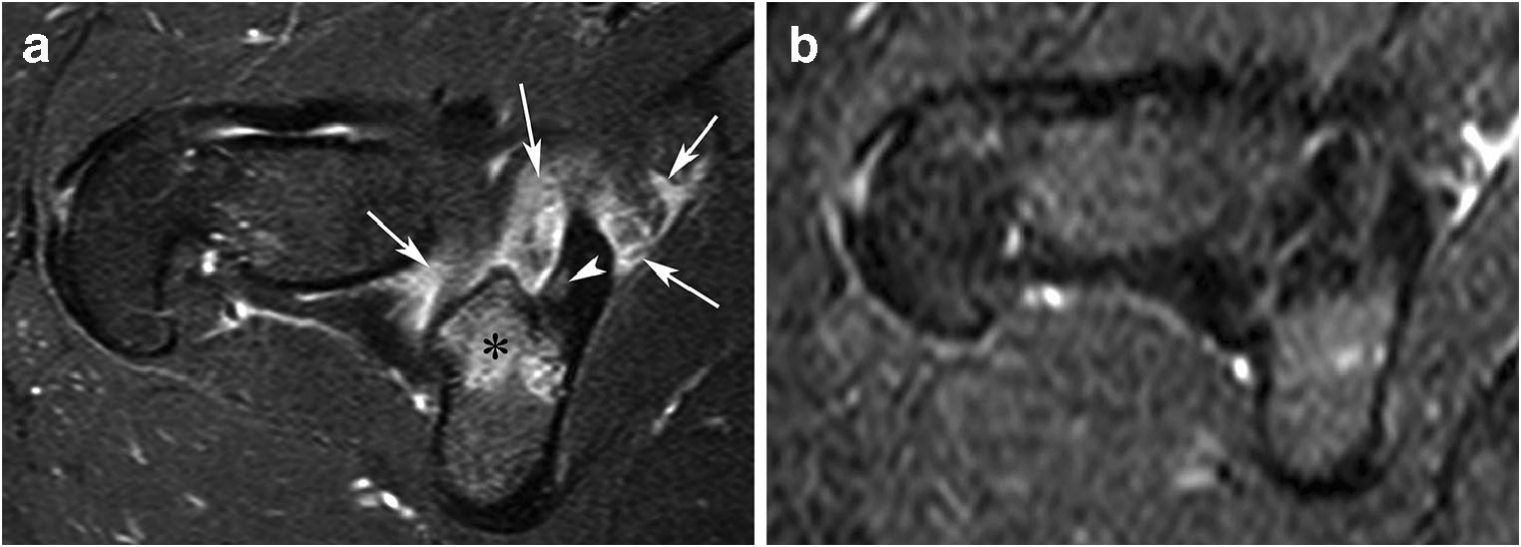
Resolution of an OO (not proved histologically) of the inferior margin of the acetabulum in a 24-year-old woman with nocturnal hip pain from several months. A bone scintigraphy showed increased focal absorption in the lower part of the acetabulum (not shown). Axial fat-saturated T2-weighted MR image (**a**) shows edema-like bone marrow in area behind the acetabulum (asterisk) and significant soft tissue edema (arrows). A small oval bone defect with intermediate signal (arrowhead) is present within a sclerotic zone of the acetabulum and corresponds presumably to an OO nidus. Because an excellent response to NSAIDs, medical treatment was proposed. Two years later, the pain had significantly decreased and an MRI showed a marked regression of the edema around the unchanged nidus (not shown). Six years later, the patient was totally asymptomatic and MRI (**b**) demonstrated complete abnormalities resolution without surgery

Epidemiology suggests that OO is self-limiting. During the 1950s, authors reported OOs that became asymptomatic after a few years [66, 67]. In 1980, Saville described a case of OO treated with aspirin and NSAIDs with complete pain resolution after 1 year and 10 months [68]. A few other published cases and small series reported complete pain resolution of NSAID-treated OOs in about half of patients after averages of 18 months to 2 years and 9 months [14, 69–71]. Resolution of OO during medical treatment can be associated in a gradual disappearance of the MRI visibility of the nidus and neighboring edema (Fig. 12).

A medical approach could be considered if the OO is clinically well tolerated with NAIDs and/or if access to ablative treatment is considered too difficult or dangerous [69–71]. Other drugs are currently investigated to treat OO including bisphosphonates which are effective in 83% of cases [72].

## Conclusion

Diagnosis and treatment of hip OO are challenging. The clinical diagnosis of hip OO is often difficult because of their deep and intra-articular location. The OO nidus can easily be overlooked leading to diagnosis of other pathologies, in particular femoroacetabular impingement or inflammatory synovitis. The use of DCE-MRI can contribute to a more accurate diagnosis of OO in particular to differentiate it from a Brodie abscess. Therapeutic approach evolved from surgical resection to percutaneous therapies and option of medical treatment remains discussed for certain specific cases.
